# The Impact of Occupational Stress on Job Burnout Among Bank Employees in Pakistan, With Psychological Capital as a Mediator

**DOI:** 10.3389/fpubh.2019.00410

**Published:** 2020-03-24

**Authors:** Arslan Khalid, Fang Pan, Ping Li, Wei Wang, Abdul Sattar Ghaffari

**Affiliations:** ^1^Department of Health Psychology, School of Nursing, Shandong University, Jinan, China; ^2^Department of Medical Psychology and Medical Ethics, School of Basic Medical Sciences, Shandong University, Jinan, China; ^3^Zhongtai Securities Institute for Financial Studies, School of Mathematics, Shandong University, Jinan, China

**Keywords:** occupational stress, job burnout, working environment, bank employees, psychological capital

## Abstract

**Background:** Job burnout is a major issue for workers in the banking sector. Many employees report feeling exhausted and want to leave their jobs due to the extra pressure and workload from their superiors and clients. They also report not being well compensated for their hard work, which they believe they do to provide the best service to their clients.

**Methods:** A cross-sectional study was made in various banks in different cities of Pakistan. An adapted questionnaire, including the effort-reward imbalance scale, psychological capital, and Maslach burnout inventory general survey were used to collect data from 1,778 male and female bank employees.

**Results:** There was a significant and positive relationship between extrinsic effort and over-commitment on the one hand, and emotional exhaustion and depersonalization on the other. It was also found that reward was negatively associated with emotional exhaustion and depersonalization. However, reward had a positive association with personal accomplishment. There was a gender difference in the mediating effect of psychological capital on stress at work and job burnout.

**Conclusion:** Male attitudes to work tend to be motivated by reward and appreciation, whereas females tend to demand a better working environment. To reduce job, burnout suitable interventions could be introduced for bank employees, whilst management support plays an important role in increasing or decreasing stress in employees.

## Introduction

Job burnout is an individual's response to emotional and interpersonal stress, which is associated with work-related stress and occupational stress (1). Job burnout is assessed by personal experience of negative continuing work stress, and the significant indicators of job-related health (2, 3). Burnout is the unpredicted outcome that causes numerous organizational problems. Burnout is omnipresent, but “the professionals who work with people have a higher risk of burnout because the sense of responsibility for human beings is greater than that of the object” (4). Job burnout has a multidimensional structure comprising emotional exhaustion, personality annihilation, and low self-efficacy (5). Job burnout can affect work efficiency, organizational satisfaction, and turnover rate (6).

In the literature analyzing the subject burnout has the three dimensions of emotional exhaustion, depersonalization and personal accomplishment (7). Burnout has been identified not only in law enforcement officers, doctors, nurses, and teachers, but also in bank employees (8, 9). The banking sector is going through an unprecedented period as a result of changes in the work organization and the global economic crisis (10). Banking is the most crucial component of the financial sector in any economy. With the opening of the banking industry, state-owned banks face stiff competition from private banks. Most employees in the banking sector are facing higher levels of stress to meet their job requirements (11). State-owned banks have recently undergone shareholding and institutional reforms, which have increased the pressure on their employees to do more work. As banks are service businesses, bank work comprises enormous interpersonal stress, which results in long-term energy depletion amongst its employees. Failure to control this burden efficiently will ultimately lead to job burnout. Therefore, it is crucial to obtain information on the status and reasons for job burnout, both to decrease the phenomenon and to prevent its influence on the work of bank employees.

To evaluate occupational stress, researchers usually apply the effort-reward imbalance (ERI) model (12). Much of the literature states that an effort-reward imbalance, together with excessive commitment can have damaging consequences such as mental illness, as well as less work satisfaction (13). Occupational stress is measured as, the critical physical and psychological response that arises when resources, skills, and needs of the workers do not match the job requirements (14, 15). The International Labor Organization emphasizes that all countries, all occupations, and all types of workers, families, and societies are influenced by occupational stress (16). Occupational stress can be seen as a product of a dynamic collaboration between the individual and the social and organizational atmosphere in which he or she works, constituting the result of the (unequal) relationship between the sources of stress associated with the task or role and the ability of the operator to respond to these issues (17). High external efforts and over-commitment can lead to emotional tiredness, depersonalization, and a decline in personal capabilities (18). Because the content of their work is related to banking employees must remain focused. Bank employees are told to pay particular attention to their appearance, especially when dealing with customers (19). Therefore, bank employees; are a vulnerable group at a high-level of risk for occupational stress. Research has recommended that banks adopt corporate policies that include stress prevention activities, with the more general goal of improving workers' mental health (20). Previous studies have pointed out that the risk factor for job burnout increases with occupational stress; occupational stress affects entire proportions of job burnout directly and indirectly (21). The risk of burnout in areas of high occupational stress is seven times greater than that in areas of low occupational stress (22).

Over the past few decades, there have been large pieces of evidence that the efficiency and productivity of any organization depend on lower levels of employee' burnout and stress at the place of work (23). Bankers face a higher level of stress because of work overload, role ambiguity, role conflicts, accountability to people, and lack of feedback (24).

The current study attempts to highlight the influence of occupational stress on employees in the banking sector and recommends that bank managers should adopt appropriate measures to develop a productive and stress-free labor force, capable of overcoming current and future challenges. Banking has become a rapidly growing service sector and therefore, a high level of staff morale is essential to deal with customers efficiently and confidently. The present study highlights suitable remedies for the specific issues of bank sector employees affected with job stress.

In recent years, researchers have tried to describe the mechanism of job burnout from a resource perspective. Psychological Capital (psyCap) is a significant individual resource that characterizes the constructive psychological state that a person develops through growth and experience (25).

It comprises four psychological resource capabilities self-efficacy, confidence, positivity, and spirit. These can be measured and use to deal efficiently with a variety of problems to produce a desirable outcome (26). In particular, employees with higher levels of PsyCap have additional resources to handle their job tasks, believe in positivity, are better able to “bounce back” rapidly from hindrances, and are extra hopeful in adverse circumstances.

Previous studies have found that PsyCap has a significant and positive relationship with an employee's professional gratification, occupational performance, managerial commitment, and worth in life (27–29). Psychological capital can be considered as a resource that averts anxiety, stress, and hopelessness (30). Furthermore, PsyCap plays the role of mediator in the association between a favorable organizational environment and employee performance (31).

The relationships between occupational stress, job burnout, and PsyCap have been analyzed in a number of different occupations (8, 32). However, these relationships have not been tested in Pakistani bank employees. The present study is focused on male and female employees in the Pakistani banking sector, and investigates the mediating role of PsyCap on the relationship between occupational stress and job burnout. We also compare job burnout, psychological capital and occupational stress among employees in the public banking sector with that of employees in the private banking sector and investigate whether there are differences in the mediating effect of PsyCap.

## Methods

### Study Design and Sample

A cross-sectional survey was conducted from March to April 2018 in Punjab Province, Pakistan (population: 110 million). Banks were randomly selected in different cities of the Punjab (North Punjab, Central Punjab, and South Punjab). As a result, the public banks were selected from the Punjab's Big five cities. The self-management questionnaire was distributed directly to 2000 employees and completed responses obtained from 1778 individuals, including 903 (50.78%) male and 875 (49.21%) female after obtaining their written informed consent and the respondent's willingness to participate.

### Demographic Characteristics

Demographic factors included age, gender, qualifications, bank type, salary, job rank, and working hours. Educational qualifications were classed as “graduate” or “higher.” The monthly salary was classed as <40000 rupees or >40000 rupees. An employee's job rank was categorized as “high official” or “low official” based on answers to questions. “Are you a high official bank employee or a regular bank employee?” The weekly working hours were divided into <45 and >45 h, based on the current standard system of working 9 h per day.

### Measures

#### Occupational Stress

The Effort-Reward Imbalance Scale (ERI) was used to define levels of occupational stress that are based on the concept of reciprocity (33). The questionnaire includes three sub-scales: 6 items for extrinsic effort (EE); 11 items for rewards (R); and 6 items for over-commitment (OC). Each response to extrinsic efforts and rewards is graded on a scale of 1 to 5, in which a higher total score indicates higher requirements for effort and higher related rewards. The response for over-commitment is proportional, ranging from 1 (demonstrating complete disagreement) to 4 (demonstrating complete agreement). Higher scores indicate a high level of job-related commitment requirements. In this experiment mean inter-item correlation was 0.26. The value of Cronbach's Alpha for the entire questionnaire reached 0.89.

### Psychological Capital (PsyCap)

PsyCap has four dimensions; (1) optimism; (2) self-efficacy; (3) resiliency; and (4) hope. These were measured by 24 items (6 items per dimension) (34). A seven-point Likert scale, ranging from one (“strongly disagree”) to seven (“strongly agree”) was used to evaluate the response. The whole psychological capital construct produced Cronbach alphas was =0.93.

### Job Burnout

Measuring job burnout used the Maslach Burnout Inventory-General Survey (MBI-GS) which has been used extensively in various occupational groups (5). The sixteen item MBI-GS questionnaire comprises three dimensions; five items for emotional exhaustion (EX); five items for depersonalization (DE); and six items for personal accomplishment (PA). There are seven possible responses to each item. These responses are scored on the stated frequency, on a range of 0 to 6. Higher scores for emotional exhaustion or depersonalization demonstrate burnout in reducing the personal accomplishment dimension to low rating (35). Research has supported the reliability of the inventory with three-factor structure and internal consistency. In this experiment Cronbach's alpha scored 0.90 in emotional exhaustion, 0.76 in personal accomplish and 0.76 in depersonalization.

### Data Analysis

(SPSS Version 21) was used to analyze data. Descriptive statistics, Pearson's correlation analysis, *t*-tests and regression analysis were conducted. Regression analysis was performed to analysis psychological capital as a mediator between occupational stress and burnout after controlling demographic factors except gender and worker-related variables such as working hours and bank type. In the mediation analysis, the value *a* denotes the association of EE, R, and OC with PsyCap, *b* the association between PsyCap and EX after directing predictive variables, *c* the association of EE, R, and OC with EX similarly. *c*′ the association of EE, R and OC with EX after adding PsyCap as a mediator; *a* × *b* is the product of *a* and *b* and the bias-corrected and accelerated C1=95%. When the *c*′ path coefficient was smaller in the second step than the *c* path coefficient in the first step or was insignificant, it was assumed that mediation might exist. Because of possible gender differences, male and female employees were analyzed separately. Statistical tests were two-tailed, and *p* < 0.05 was considered as statistically significant.

### Ethics

Approval for the study was obtained from the Institutional Ethics Committee of the School of Basic Medical Sciences, Shandong University.

## Results

As shown in Table 1, the average age of the respondents was 42.59 ± 9.72 years. The average extrinsic effort level of respondents was 18.05 ± 3.46; the average reward level was 32.97 ± 4.87; the average over-commitment level was 14.99 ± 2.72; the average psychological capital level was 84.29 ± 8.48; and the average emotional exhaustion level was 15.17 ± 4.35; the average depersonalization level was 15.09 ± 4.46; and the average personal accomplishment level was 18.01 ± 4.82.

**Table 1:** Descriptive statistics of all variables.

**Individual factors**	**Category/Minimum**	**Frequency (%)/Maximum**	**Mean**	***SD***
Gender	Male	903 (50.79)	–	–
	Female	875 (49.21)	–	–
Qualification	Graduate	879 (49.44)	–	–
	Higher	899 (50.56)	–	–
Salary (Rupees)	Up to 40000	884 (49.72)	–	–
	Above 40000	894 (50.28)	–	–
Job rank	High official	628 (35.32)	–	–
	Low official	1150 (64.68)	–	–
Working hours (Daily)	Up to 9	591 (33.24)	–	–
	Above 9	1187 (66.76)	–	–
Bank type	Public (No. of employees)	481 (27.05)	–	–
	Private (No. of employees)	1297 (72.95)	–	–
Age	26	59	42.59	9.72
Extrinsic effort	7	29	18.05	3.46
Reward	20	46	32.97	4.87
Overcommitment	7	23	14.99	2.72
PsyCap	58	116	84.29	8.48
Emotional exhaustion	3	29	15.17	4.53
Depersonalization	0	28	15.09	4.46
Personal accomplishment	4	33	18.01	4.82

Table 2 indicates the correlations between the study variables. The findings reveal that both extrinsic effort and over-commitment had a significant and negative correlation with reward, whereas the relation between over-commitment and extrinsic effort was significant and positive. Psychological capital had a significant and negative correlation with extrinsic effort and over-commitment but a significant and positive correlation with reward. The relation of emotional exhaustion with reward and psychological capital was significantly negative whereas significantly positive with extrinsic effort and over-commitment. Similarly, depersonalization was significantly negatively correlated with reward and psychological capital but significantly positively correlated with extrinsic effort, over-commitment and emotional exhaustion. Moreover, personal accomplishment had a significant and negative correlation with extrinsic effort, over-commitment, emotional exhaustion and depersonalization together with a significant positive correlation with reward and psychological capital.

**Table 2:** Correlation between occupational stress, psychological capital and job burnout.

**Variables**		**M ± SD**	**EE**	**R**	**OC**	**PC**	**EX**	**DE**	**PA**
Occupation stress	EE	18.05 ± 3.46	1						
	R	32.97 ± 4.87	−0.774^**^	1					
	OC	14.99 ± 2.72	0.753^**^	−0.612^**^	1				
Psychological capital	PsyCap	84.29 ± 8.48	−0.793^**^	0.653^**^	−0.900^**^	1			
Job burnout	EX	15.17 ± 4.53	0.923^**^	−0.808^**^	0.737^**^	−0.789^**^	1		
	DE	15.09 ± 4.46	0.618^**^	−0.774^**^	0.485^**^	−0.530^**^	0.650^**^	1	
	PA	18.02 ± 4.82	−0.872^**^	0.859^**^	−0.678^**^	0.727^**^	−0.910^**^	−0.684^**^	1

EE, Extrinsic Effort; R, Reward; OC, Over-commitment; EX, Emotional Exhaustion; DE, Depersonalization; PA, Personal Accomplishment. *Significant at the 0.05 level (2-tailed);

** *significant at the 0.01 level (2-tailed)*.

Table 3 shows the results of regression analysis with psychological capital as the mediator variable and emotional exhaustion as an outcome variable, with the aim of investigation the influence of psychological capital relationship between extrinsic effort, over-commitment, and reward (as independent variables) with emotional exhaustion (dependent variable).

**Table 3:** Regression analysis, using psychological capital as a mediator and emotional exhaustion as an outcome.

**Predictors**	**Path coefficients**				a ^*^ b (95% CI)	***R^**2**^***
	***a***	***b***	***c***	***c^**′**^***		
**Male**						
Extrinsic effort	−0.943^***^	−0.496^***^	0.884^***^	0.416^***^	0.468 (0.163, 1.426)	0.807
Reward	0.606^***^	−0.774^***^	−0.658^***^	−0.189^***^	−0.469 (−0.533, −0.315)	0.811
Over-commitment	−0.955^***^	−0.702^***^	0.865^***^	0.195^**^	0.670 (0.720, 1.334)	0.792
**Female**						
Extrinsic effort	−0.656^***^	−0.114^***^	0.963^***^	0.888^***^	0.075 (0.035, 0.250)	0.935
Reward	0.700^***^	−0.036^**^	−0.969^***^	−0.944^***^	−0.025 (−0.154, 0.025)	0.940
Over-commitment	−0.851^***^	−0.621^***^	0.617^***^	0.089	0.528 (0.670, 1.000)	0.486

** significant at the 0.01 level (2-tailed);

*** *significant at the 0.001 level (2-tailed)*.

For male bank employees, extrinsic effort and over-commitment were negatively associated with psychological capital, whereas reward was positively associated with psychological capital (from path *a*). By controlling the predictor variables, psychological capital was significantly and negatively associated with emotional exhaustion (from path *b*). As for indirect effects, extrinsic effort and over-commitment were significantly and positively associated with emotional exhaustion, whereas reward was negatively associated with emotional exhaustion (from path *c*). When psychological capital was involved in the model as a mediator, the direct pathway between extrinsic effort and emotional exhaustion remained statistically significant, whereas that between reward was not statistically significant (from path *c*′).

For female bank employees, extrinsic effort and over-commitment were negatively, associated with psychological capital whereas reward was positively associated with psychological capital (from path *a*). On the indirect effects, extrinsic effort and over-commitment were significantly and positively associated with emotional exhaustion, whereas reward was negatively associated with emotional exhaustion (from path *c*). Consequently, psychological capital mediated significantly the associations of extrinsic effort, reward, and over-commitment with emotional exhaustion. When psychological capital was involved in the model as a mediator, the direct pathways between reward, over-commitment, and emotional exhaustion (from path *c*′) became significant.

To evaluate the effect of the mediating pathway, the proportion we calculate of the total effect of occupational stress on job burnout by psychological capital as a mediator using the formula *(a* × *b)/c*. The proportions of psychological capital mediation for male employees were 44.8% for extrinsic effort, 46.9% for reward, and 67.0% for over-commitment. The proportions of psychological capital mediation for female employees were 7.5% for extrinsic effort, 2.5% for reward, and 52.8% for over-commitment.

Table 4 shows the results of regression analysis with psychological capital as the mediator variable and depersonalization as an outcome variable, with the aim of investigation the influence of psychological capital on relationship between over-commitment, extrinsic effort, and reward (the independent variables) and depersonalization (as the dependent variable). The results showed positive associations of reward with depersonalization, and a negative association of extrinsic effort, and over-commitment, with depersonalization, between both female and male bank employees (from path *a*). The mediating role of psychological capital in the relationship among occupational stress and depersonalization was then assessed. A negative association with reward, extrinsic effort, and over-commitment was observed among both male and female bank employees (from path *b*). From path *c*′ we observed positive association with extrinsic effort and over-commitment, and a negative association with reward among both male and female bank employees (indirect effect).

**Table 4:** Regression analysis, using psychological capital as a mediator and depersonalization as an outcome.

**Predictors**	**Path coefficients**				a ^*^ b (95% CI)	***R^**2**^***
	***a***	***b***	***c***	***c^**′**^***		
**Male**						
Extrinsic effort	−0.943^***^	−0.695^***^	0.417^***^	−0.238^**^	0.655 (0.296, 1.750)	0.225
Reward	0.606^***^	−0.036	−0.737^***^	−0.715^***^	−0.022 (−0.039, 0.001)	0.543
Over-commitment	−0.955^***^	−0.506^***^	0.445^***^	−0.038	0.483 (0.471, 1.061)	0.219
**Female**						
Extrinsic effort	−0.656^**^	−0.081^***^	0.824^***^	0.771^***^	0.053 (0.042, 0.099)	0.682
Reward	0.700^***^	−0.036	−0.812^***^	−0.787^***^	−0.025 (−0.044, −0.009)	0.659
Over-commitment	−0.851^***^	−0.511^***^	0.524^***^	0.089	0.435 (0.558, 0.842)	0.345

** Significant at the 0.01 level (2-tailed);

*** *Significant at the 0.001 level (2-tailed)*.

Thus, psychological capital significantly mediated the associations of extrinsic effort, reward, and over-commitment with depersonalization. When psychological capital was involved in the model as a mediator, the direct pathway between extrinsic effort, and psychological capital remained statistically significant. To evaluate the effect size of the mediating pathway, the proportion we calculated of the total effect of the independent variable on the dependent variable by psychological capital using the formula *(a* × *b)/c*. The proportions of psychological capital mediation for male employees were 65.5% for extrinsic effort, and 48.3% for over-commitment. The proportions of psychological capital mediation for female employees were 5.3% for extrinsic effort, and 43.5% for over-commitment.

Table 5 shows the results of regression analysis with psychological capital as the mediator variable and personal accomplishment as the outcome variable. The results shows a positive associations of reward with personal accomplishment and a negative association of extrinsic effort and over-commitment with personal accomplishment among both male and female bank employees (from path *a*). From the path *b*, positive associations with reward, extrinsic effort, and over-commitment were observed among the male employees whereas a positive association with reward and negative association with extrinsic effort and over-commitment were determined among the female employees.

**Table 5:** Regression analysis, using psychological capital as a mediator and personal accomplish as an outcome.

**Predictors**	**Path coefficients**				a ^*^ b (95% CI)	***R^**2**^***
	***a***	***b***	***c***	***c′***		
**Male**						
Extrinsic effort	−0.943^***^	0.619^***^	−0.755^***^	−0.171^**^	−0.584 (−1.624, −0.170)	0.611
Reward	0.606^***^	0.530^***^	0.735^***^	0.414^***^	0.321 (0.198, 0.437)	0.717
Over-commitment	−0.955^***^	0.562^***^	−0.765^***^	−0.229^**^	−0.537 (−1.262, −0.463)	0.613
**Female**						
Extrinsic effort	−0.656^***^	0.044^***^	−0.993^***^	−0.964^***^	−0.029 (−0.066, −0.021)	0.988
Reward	0.700^***^	−0.039^***^	0.995^***^	0.102^***^	−0.027 (−0.038, −0.020)	0.991
Over-commitment	−0.851^***^	0.620^***^	−0.594^***^	−0.067	−0.528 (−1.043, −0.678)	0.458

** Significant at the 0.01 level (2-tailed);

*** *Significant at the 0.001 level (2-tailed)*.

From the path *c* negative associations with extrinsic effort and over-commitment and a positive association with reward were observed between both female and male employees.

To evaluate the effect size of the mediating pathway, the proportion we calculate of the total effect of the independent variable on the dependent by psychological capital using the formula *(a* × *b)/c*. The proportions of psychological capital mediation for male employees were 58.4% for extrinsic effort, 32.1% for reward, and 53.7% for over-commitment. The proportions of psychological capital mediation for female employees were 2.90% for extrinsic effort, 2.7% for reward, and 52.8% for over-commitment.

Table 6 presents a comparison of mediator and outcome variables between employees in the public and private banking sectors. We conclude that extrinsic effort, reward, over-commitment, psychological capital, emotional exhaustion and personal accomplishment were the statistically significant differences between employees in the public and private sector of banking whereas depersonalization did not differ significantly in this research. The extrinsic effort-reward ratio was the statistically significant higher between employees in the private sector of banking than that of employees belongs to public banking sector.

**Table 6:** Comparison of occupational stress, psychological capital, and job burnout between public and private banking sector employees.

**Variables**		**Public sector bank (*n* = 481)**	**Private sector bank (*n* = 1297)**	***T***	***P***
Occupational stress	Extrinsic effort	17.51 ± 3.70	18.24 ± 3.35	−4.003	<0.000
	Reward	33.32 ± 5.28	32.83 ± 4.70	1.878	<0.030
	Extrinsic effort/Reward ratio (Rationalized)	1.01 ± 0.34	1.06 ± 0.0.34	−3.040	<0.001
	Over–commitment	14.70 ± 2.79	15.09 ± 2.69	−2.727	<0.003
Psychological capital	PsyCap	85.28 ± 8.69	83.93 ± 8.37	2.993	<0.002
Job burnout	Emotional exhaustion	14.33 ± 4.79	15.48 ± 4.39	−4.749	<0.000
	Depersonalization	14.84 ± 4.95	15.18 ± 4.26	−1.415	0.079
	Personal accomplish	18.51 ± 5.24	17.83 ± 4.65	2.621	<0.007

## Discussion

A workplace is a place where individuals spend a major portion of the day, and as the workplace seeks more demands, creates greater responsibilities, and induces uncertainty, there is an increasing risk of stress for the workforce (36). All professional groups face stress in their jobs including bankers. Stress acts as a threat physiologically and psychologically to better employee performance, and studies have shown that occupational stress plays a vital role as a risk factor for employee burnout.

Occupational stress affects all dimensions of burnout directly and indirectly (12, 21, 37). The risk of burnout in areas of high occupational stress is seven times greater than that in areas of low occupational stress (22).

The findings of our study highlight the correlation between occupational stress and job burnout. Reward was found to be positively related to personal achievement whereas extrinsic effort and over-commitment were found to be negatively related to personal achievement. Alternatively, reward was found to decrease exhaustion and depersonalization whereas extrinsic effort and over-commitment increased the risk of job burnout (38). Our findings are consistent with previous studies that have identified negative associations between reward and emotional exhaustion and between reward and depersonalization; whereas extrinsic effort and over-commitment have a positive relation with those burnout dimensions (emotional exhaustion and depersonalization). This could be because over-commitment and extrinsic effort entail continuous work, and much energy can be dissipated this way hence derailing the achievement of goals and fulfillment of the customer's requirements.

To overcome these problems, research has suggested interventions to curb occupational stress via a prevention plan and depression treatment for employees (36). These interventions include communication between management and staff and the management of employee workload. In this way, employees should avoid over-commitment by effective time management. In particular, research has suggested interventions for banks employees, following cross-sectional study of employees in Chinese banks (39).

According to Tsai et al. (40), exercise is the best way to reduce job stress and alleviate metabolic problems. Exercise has many positive effects that can reduce the impact of job demands, and can significantly reduce the risk of work-related burnout. Management support also plays an important role in increasing or decreasing stress in employees (24).

In support of the findings of previous research (21, 37), our study found that occupational stress had an impact on burnout in public and private bank employees through the mediating effects of Psychological Capital (PsyCap). PsyCap can be affected by individual factors. Our study identified a negative association between PsyCap and over-commitment and also showed that PsyCap had the positive effect of reducing the negative impact of occupational stress. By improving PsyCap, we can reduce the risk of stress and burnout among employees. Studies have found that PsyCap has a significant and positive association with an employee's job satisfaction, organizational commitment, job performance, and life quality (27–29, 41–43). To improve PsyCap for bank staff in Pakistan, some interventions could be implemented such as those mentioned above; bank managers should communicate with employees, establish neutral and fair system, and improve the personal qualities of their staff through enhanced training programs.

It is noticed that our research on the banking sector in Pakistan identified a gender difference in the mediating effect of PsyCap on occupational stress and job burnout. The reasons might be explained that the male and female employees responded differently according to their attitudes toward work, which reflects a difference in their requirements. The male employees tend to be motivated by the reward and appreciation, whereas, the female employees tend to demand a better working environment. The results were supported by a negative association between PsyCap and occupational stress among female employees whereas this was not the same for males (36).

This study also compared the private and public banking sector in Pakistan, in term of stress among employees and the other variables. It was found that the private banks provided better pay and rewards to motivate their employees. In return, however they also place a heavier burden of work and responsibilities on their shoulder which can be difficult to carry. Although, they have a better working atmosphere, they also face the pressure of work from their superiors and customers, which can result in stress at the workplace. Bankers generally face a higher level of stress because of work overload, role ambiguity, role conflicts, accountability to people, and lack of feedback (24).

In contrast employees in public banking can have better job security. Many have a permanent contract and knowledge that they will not be sacked if they commit some irregularity at work. They receive fixed salary, and not extra rewards are provided for their performance level. Hence, there is no difference in their income level, whether they work hard or not, which can also result in job burnout. Previous research found that an effort-rewarding imbalance, together with excessive commitment, is a predictor of negative consequences such as mental illness, and immune dysfunction, as well as a low level of job satisfaction (13, 44).

## Limitation of the Study and Future Recommendations

As well as the significant contributions of the study, there are also some limitations which should be noted. The first is the study was focused on employees in the banking sector, which limits generalizations arising from findings. Second, a cross-sectional design used to creating it difficult to draw any casual relationships between occupational stress, job burnout and PsyCap. Additionally data were obtained through self-report, which can be a source of bias. Employees may have overestimated or underestimated the association among occupational stress and job burnout. Therefore, findings should be interpreted with prudence. Future research could samples of employees working in a range of sectors to assess generalizability of our study. Future research could also investigate employees working in other countries, adopting same model to investigate results.

PsyCap was taken as a mediator in the relationship between organizational stress and job burnout, and could be replaced by other variables in future studies. Also, only three components of organizational stress were investigated: namely extrinsic effort, over-commitment, and reward future studies could investigate; other components of organizational stress either alongside the three components or replacing them.

Similarly, only three component of job burnout were investigated namely: emotional exhaustion; depersonalization; and personal accomplishment. Future studies, could investigate, other components of job burnout alongside or replacing those three components.

## Conclusion

The banking sector in Pakistan makes a large contribution to its economy, but rising job burnout due to occupational stress can be a barrier to economy growth. Extrinsic effort and over- commitment results in emotional exhaustion and depersonalization with a negative impact on PsyCap and personal accomplishment. However, the reward results in a decrease in emotional exhaustion and depersonalization and has positive impact on PsyCap and personal accomplishment. Both public and private bank employees exhibit different dimensions of occupational stress, including extrinsic effort, reward, and over-commitment. Those effects can be reduced by improving PsyCap which would also decrease the risk of burnout.

## Data Availability Statement

The datasets generated for this study are available on request to the corresponding author.

## Ethics Statement

Approval for the study was obtained from the Institutional Ethics Committee of the School of Basic Medical Sciences, Shandong University. The patients/participants provided their written informed consent to participate in this study.

## Author Contributions

AK performed major work and wrote the manuscript. PL, WW, and AG provided the support for complete the manuscript. FP revised the paper.

### Conflict of Interest

The authors declare that the research was conducted in the absence of any commercial or financial relationships that could be construed as a potential conflict of interest.
